# Supplementary material for: Content-rich biological network constructed by mining PubMed abstracts
Source: BMC Bioinformatics. 2004 Oct 8;5:147. doi: 10.1186/1471-2105-5-147 (PMC528731; doi:10.1186/1471-2105-5-147)
Supplement: Additional File 5 — The original Chilibot query results of the term "long-term potentiation (LTP)" and 22 other terms, limiting the latest references analyzed to the years 1990, 1995, 2000, and 2004. [file 1471-2105-5-147-S5.bz2 › chilibotAdditionalFile5/ltp2000/html/PLC_CAMKII.html]

 


 **PLC** and **CAMKII** 
  
Found 2 abstracts in PubMed,  **2 abstracts were retrieved and analyzed**.  


---

 Search Google  |
 PDF files only 
|  EDU domain only 

---

**Interactive relationship** (e.g. stimulation, inhibition, etc)

- It is thus concluded that InsP3 3 kinase up regulation occurs through activation of  **PLC**  coupled serotoninergic receptors and requires the phosphorylation of the enzyme by the ubiquitous multimeric protein kinase  **CaMKII** .  Ref: 9835216 Neurosci Lett, 1998

- :-)

  **Parallel relationship** (e.g. studied together, co-existance, homology, etc.)

  - Also, the release of CRF depends on the  **PLC**  beta to InsP3 R axis and  **CaMKII**  activity but also entails the participation of a calcium independent PKCs.  Ref: 10919265 Endocrinology, 2000
  - The stimulation of CRF synthesis by NPY seems to depend mainly on a  **PLC**  beta to InsP3 R axis and on  **CaMKII**  activity.  Ref: 10919265 Endocrinology, 2000
